# Linking Urban Planning, Community Environment, and Physical Activity: A Socio-Ecological Approach

**DOI:** 10.3390/ijerph20042944

**Published:** 2023-02-08

**Authors:** Xue Zhang, Mildred E. Warner

**Affiliations:** 1 Lerner Center for Public Health Promotion and Population Health, Center for Policy Research, Syracuse University, Syracuse, NY 13244, USA; 2 Department of City and Regional Planning, Cornell University, Ithaca, NY 14853, USA

**Keywords:** physical activity, socio-ecological model, urban planning, cross-agency collaboration, community policy

## Abstract

Lack of physical activity is a growing concern among public health advocates and urban planners. Our socio-ecological model incorporates urban planning and World Health Organization actions on physical activity to identify key factors related to leisure-time physical activity at the community level. Our 2019 nationwide US survey of 1312 communities enables examination of the influence of individual, community, and policy levels on physical activity. Individual factors—poverty, aging, minority population, and longer commuting time—result in lower physical activity. Community-level factors have both positive and negative effects. Physical activity is lower in rural and suburban communities, but higher in communities with more transportation services, recreation and social activities, and safety. Communities with mixed-use neighborhoods and complete streets also show higher levels of physical activity. At the policy level, zoning and cross-agency collaboration have an indirect effect on physical activity by increasing these community-level factors. This suggests an alternative approach to promoting physical activity. Local governments can promote transportation, recreation and safety, especially in rural and minority communities lacking active-friendly built environments and facing challenges from aging population, poverty, and longer commuting time. This socio-ecological approach can assess multilevel factors related to physical activity in other countries.

## 1. Introduction

Regular physical activity could prevent non-communicable diseases and improve quality of life [1]. However, globally, 25% of adults do not meet the World Health Organization’s (WHO) standard of physical activity [1]. Across US counties, on average 27% of people have no leisure time physical activity, and this ranges from 9.5% to 50% [2]. Compared to urban counties, suburban and rural counties have a significantly higher percentage of the population that is not physically active (urban county = 24%, suburban county = 27%, rural county = 28%), has limited access to exercise opportunities (urban county = 16%, suburban county = 37%, rural county = 42%), and is obese (urban county = 30%, suburban county = 33%, rural county = 33%) [2].

To improve physical activity, “The Physical Activity Guidelines for Americans” from the Centers for Disease Control (CDC) recommends that the built environment of communities should support active transportation with more people walking and biking [3]. However, those recommendations are difficult for suburban and rural communities to implement, as many of those communities lack mixed-use and walkable neighborhoods [4,5], and residents are more likely to rely on automobiles [6]. Additionally, there are no consistent results on the relationship between the built environment and physical activity [7,8]. For rural communities, research shows that people living in small towns walk more for recreational purposes, and the slow traffic speed plays a more important role [6].

Both public health and urban planning need to look beyond the built environment and pay attention to the social and policy environment. WHO’s [1] actions on physical activity emphasize that transportation services, safety, and recreation programs are important community features to promote an active society. The policy environment, such as community planning and cross-agency collaboration, are important factors contributing to those community features [9,10,11]. Creating an active-friendly environment requires a multi-level socio-ecological approach with local policies targeting social, physical, and environmental determinants of health that support physical activity [8,12,13]. This study contributes to the growing research on the impact of the community environment on physical activity, by giving attention to transportation services, recreation programs and perceptions of safety, as well as urban planning and cross-agency collaboration.

To empirically examine factors that contribute to a higher level of physical activity at multiple levels in the socio-ecological model, we conducted a 2019 national survey of US local government officials to measure governments’ actions at the policy level (planning and zoning) and community level (e.g., built environment, services, safety). We linked the survey data with data on individual-level leisure time activity from Centers for Disease Control and Prevention: Population Level Analysis and Community Estimates (CDC: PLACE) [14], and demographic data from American Community Survey [15]. We ran a structural equation model to link the individual, community and policy levels and simultaneously estimate the relation between the effects of urban planning, community environment and demographic factors on physical activity. We were interested in addressing the following questions. How does the policy level in the socio-ecological model interact with the community level to promote physical activity? What role does planning policy play? Which demographic and community factors matter most for physical activity?

This study makes three contributions. First, drawing from WHO’s physical activity guidelines, it develops measures of the social and physical environment in the socio-ecological model, including mixed-use neighborhoods, complete streets supporting walking and biking, transportation services, recreation and social activities, and perceptions of safety. Second, it measures the roles of urban planning in the policy environment, including neighborhood and street zoning codes, and cross-agency collaboration among planning, public health, transportation, and parks and recreation departments. Third, it uses structural equation modeling to make explicit linkages between individual, community and policy levels in the socio-ecological model that are related to a higher level of physical activity.

## 2. Literature Review

The socio-ecological model integrates behavior theories and social ecology to explain determinants of physical activity at the individual, social, environmental and policy levels [7,12]. The socio-ecological model emphasizes the importance of community environment and policy in health behaviors, rather than solely focusing on individual characteristics [12,13]. It shows the importance of considering individual, community, and policy layers as a holistic system to improve physical activity. For example, interventions in physical activity at the individual level are less likely to be effective when the physical and social environment create barriers to an active lifestyle (e.g., unsafe neighborhoods, lack of recreation facilities) [16,17]. Interventions in the community environment and policy levels are viewed as the most promising strategy to improve physical activity [13].

Although the socio-ecological model pays attention to various determinants of health at different levels, more studies are needed to understand the impact of the social environment on health behaviors [18,19]. The physical and social environment, where people live, work and play, are important social determinants of health [20,21]. Most studies show that a mixed-use neighborhood, public transportation, and complete streets that support walking and biking could encourage pedestrian travel and physical activity [22,23,24]. The social environment and recreation facilities also play an important role in increasing physical activity [7,12,24,25]. Neighborhood safety is related to people’s mobility patterns, especially for older adults [26]. In rural communities, safety in streets, parks and playgrounds has a direct impact on physical activity [27,28,29]. 

One challenge in the socio-ecological model is how to link policy factors with other determinants. Policy is considered an “upstream factor” in the socio-ecological model, as it could affect the whole population [7,30]. Few studies directly consider the impact of community policy on physical activity. Addressing health disparities requires a multi-level analytical approach [16,30], but the linkage between policy and other levels is understudied. The policy intervention needs to fit with the social and physical environment of local communities, as the sociocultural structure could lead to the intervention being less effective [16].

This study fills the research gap in the ecological model by integrating the role of urban planning at the community policy level. Urban planners focus on issues related to land use, transportation and environmental safety to improve public health [13]. Historically, zoning codes separated land use into residential, commercial, and industrial to protect public health [31]. With the aging population, zoning codes have shifted to new urbanist neighborhood designs that promote mixed land use, transit-oriented design and pedestrian-oriented zoning to encourage more walking and less driving [32,33] and address both gender and aging concerns [34]. A nationwide US local government survey found that when planning and community design are focused on street walkability and mixed use, communities have more sidewalks, bike lanes, parks and playgrounds, public gathering spaces, etc. [35]. However, rural communities lag in these built environment features [4,36,37]. Thus, efforts to promote physical activity in rural communities often focus more on recreation than on active transportation [5,6].

Policies often require partnerships beyond the health sector to influence the physical environment and promote physical activity [7]. Improving collaboration between public health and urban planning is emphasized by WHO [1] and the American Planning Association [32]. Cross-agency collaboration is recognized by collective impact theory as key to addressing health disparity [16]. Cross-agency collaboration affects the policy and institutional layer, and has been found to be important in building healthy places for children and older adults [9,11,38,39]. The Robert Wood Johnson Foundation [40] emphasizes the importance of cross-sector collaboration to identify solutions to improve well-being and build a culture of health. Land use policy, planning, transportation, and physical activity are closely related, so collaboration between planners, transportation and health researchers and policymakers can help create environments that promote physical activity [41].

**Figure 1:**
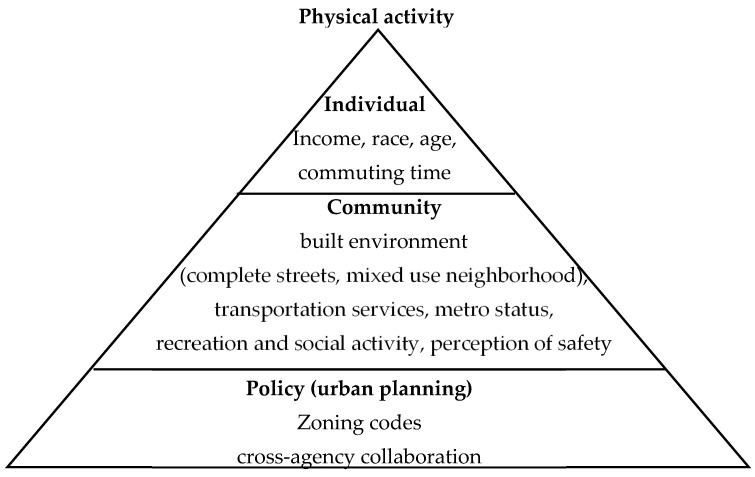
Theoretical framework. Data source: Author analysis.

This study uses the socio-ecological model to estimate key factors at the individual, community, and policy levels that are related to physical activity (Figure 1). At the policy level, urban planning literature identifies the role of zoning [32,37] and cross-agency collaboration [9,16,38,39] in promoting community health. At the community level, research shows that physical activity is related to community features, including mixed-use neighborhoods, transportation services, complete streets [22,23,24], recreation and social activity [7,12,24,25], perceptions of safety [27,28,29], and metro status [5,6,39]. We use demographic structure to capture the individual level, including income, race, age, and commuting time. Our theoretical framework assesses multilevel and multidimensional factors related to physical activity (Figure 1).

## 3. Materials and Methods

### 3.1. Study Sample

To assess the relation between physical activity and the levels of individual, community, and policy (Figure 1), we developed a national-level dataset including the recent health data on leisure-time physical activity from the CDC 500 Cities & PLACE data portal (updated in January 2021) [14], and linked this with a 2019 nationwide survey on the community-level physical, social and policy environment. We also include data on demographic structure from the American Community Survey (2015–2019) [15].

Our nationwide US survey data measure social, physical, and policy factors related to building a healthy community. We collaborated with the International City/County Management Association (ICMA) to send the Planning for All Ages survey to city and county managers across the US in 2019. The survey sample frame included all counties and all municipalities with over 25,000 population, a one-in-three sample of municipalities with under 25,000 population, and a one-in-2.5 sample of towns and townships with over 2500 in population for a total of 8016 local governments. Of that total, 1312 municipalities responded, for a response rate of 16%. A two-sample Kolmogorov–Smirnov test showed that the sample was representative by geography but captured more places larger than rural communities. Rural communities represented 31% of the sample, which enabled us to look at rural–urban differences.

The survey explored physical environment features including complete streets (streets designed for walking and biking), mixed-use neighborhoods (e.g., a mix of retail, services, and housing), transportation services (e.g., public transit), and social and community context (e.g., perceptions of safety, recreation programs and social activities) [42]. This survey also measured local government actions to build a healthy community in terms of planning, zoning, and cross-agency collaboration. This comprehensive dataset allowed us to examine key factors at different levels of the socio-ecological model and the relation between urban planning and the community physical and social environment.

### 3.2. Variables

#### 3.2.1. Physical Activity

Our primary dependent variable of interest was leisure-time physical activity, which is the reverse-coded measure of the crude prevalence of no leisure-time physical activity among adults aged ≥18, from the CDC 500 Cities & PLACES Data Portal [14]. The data were acquired from the Behavioral Risk Factor Surveillance System (BRFSS) question “During the past month, other than your regular job, did you participate in any physical activities or exercises such as running, calisthenics, golf, gardening, or walking for exercise?” The CDC 500 Cities & PLACES Data Portal dataset contains model-based estimates. Data sources included the Behavioral Risk Factor Surveillance System (BRFSS) 2018 or 2017 data, Census Bureau 2010 population estimates, and American Community Survey (ACS) 2014–2018 or 2013–2017 estimates. The CDC released three levels of measures: county level, place level, and census tract level.

To match our nationwide survey data, we used the physical inactivity data at the census tract level to construct the data at the level of county subdivision (e.g., city, town, village). County subdivision data were weighted by the intersecting area between the county subdivision and each census tract.

$$\mathrm{County}\;\mathrm{subdivision}\;\mathrm{data}=\sum_{i=1}^{n}w_{i\;}Data_{i}$$

where *w_i_* is the weight of census tract *i*, *Data_i_* is the data of census tract *i*.

$$w_{i}=\frac{Area_{i}}{\sum_{i=1}^{n}Area_{i}}$$

where *Area_i_* is the intersecting area between county subdivision and census tract *i*. 

Descriptive statistics of model variables are shown in Table 1. Table 1 shows that on average, 76% of the population participated in physical activities or exercise during the past 30 days other than their regular jobs.

#### 3.2.2. Individual Level: Demographic Factors

Income, age, race, and time are important determinants of the level of physical activity. These demographic factors capture the individual features of a community. Demographic factors include poverty rate, percentage of the minority population and percentage of the population over age 65. We also included average commuting time to work, as this would reduce time available for physical activity. Data were drawn from the American Community Survey estimates (2015–2019) [15]. We expected less physical activity in communities with lower income, older populations, more minorities and higher commuting.

#### 3.2.3. Community Level

We were interested in the physical and social factors at the community level that are related to a higher level of physical activity. Community measures, drawn from our theoretical framework, included: built environment (complete streets, mixed-use neighborhoods), transportation services, recreation and social activity, perceptions of safety, and metro status. These variables formed the other dependent variables in our set of structural equation models.

##### Built Environment

We developed indices composed of several metrics for complete streets and mixed-use neighborhoods, following prior research [35]. Each built environment metric was assessed on a scale of 1 to 5 for percentage of community covered (0% = 1, 1–25% = 2, 26–50% = 3, 51–75% = 4, >75% = 5). For example, “1” indicated that a community did not have this built environment feature, and “5” meant more than 75% of the community had the built environment feature. We expected that built environments that support active transportation would be related to more physical activity.

The complete streets index is the sum of three built environment metrics: the percentage of the community covered by (1) sidewalk systems, (2) bike lanes, and (3) streets designed for all modes of transit—walking, biking, etc. - not only cars. As a result, the complete streets index was on a scale of 3 to 15 for each community (Table 1). A higher number meant the community had more complete street features that support walking and biking. Table 2 shows the components of the complete streets index. Sidewalk systems connecting residents and services were the most common feature (Table 2). Almost 40% of responding communities reported that more than half of their community was covered by a sidewalk system. However, bike lanes and streets designed for all modes of transit were not common. About a third of communities lacked those features, and only a small fraction reported that more than half of their community had bike lanes (7%) or streets designed for all modes of transit (14%).

The mixed-use neighborhood index is the sum of four built environment metrics: the percentage of the community covered by: (1) parks or playgrounds, (2) public spaces, (3) a mix of retail, services and housing, and (4) fresh food markets. The mixed-use neighborhood index was on a scale of 4 to 20 for each community (Table 1). A higher number meant the community had more mixed-use neighborhood features. Compared to the complete streets index, mixed-used neighborhood features were more common in communities (Table 2). Thirty-nine percent of communities reported more than half of their community had access to parks or playgrounds within a half-mile of residents. Public gathering spaces were less commonly reported, and only 39% of communities reported these in more than half of their community. Mixed-use included a mix of retail, services, and housing, but only 29% of respondents reported that more than half of their community was characterized by mixed use. Fresh food markets were more likely to be concentrated in one part of the community. Forty-seven percent of communities reported that less than a quarter of their neighborhoods had a fresh food market.

##### Transportation Services

Mobility-related services provided by the community included four elements. Just over half of communities had public transit (57%) and demand–response transit (aka “dial-a-ride”, 56%). The lack of public transit services was due in part to the rural communities in the sample. Only about a fifth of communities had volunteer driver programs (21%). The least common element comprised communities where school buses were used to transport seniors (11%). We expected that more transportation services could facilitate physical activity.

##### Recreation and Social Activity

Recreation and social activity were measured by the number of programs supported by local government. Among the four elements measured, recreation programs were the most common. About 60% of communities had recreation programs. Other elements included social activities (44%), community gardens (39%), and checking on your neighbors (22%). More recreation and social activities were expected to be associated with a higher level of physical activity.

##### Perceptions of Safety

Perceptions of safety were measured by two questions in the survey. Each question was measured on a scale of 1 (strongly disagree) to 5 (strongly agree). More than 70% of respondents agreed that "crimes rates are low in my community" and "residents feel safe and secure in streets and parks". A safe neighborhood was expected to be associated with a higher level of physical activity.

##### Metro Status

Communities were grouped into metro core, suburbs, and rural areas based on US Census delineations [43]. Metro core places had at least one principal city, and suburbs were other places inside metropolitan areas. Rural areas were nonmetropolitan places and constituted 31% of the sample. Metro core places were set as the reference.

Community controls included population and population density from the American Community Survey (2015–2019). Community controls also included the median age of housing to capture older communities that may be more walkable. We expected that larger, denser, and older places may be more likely to have walkable built environment features.

#### 3.2.4. Policy Level: Planning, Zoning and Cross-Agency Collaboration

We used urban planning to measure the policy environment in the socio-ecological framework, including zoning codes and the level of cross-agency collaboration among public health and urban planning agencies. We linked the urban planning measures with the community’s social and physical environment. We expected that an active living environment would be related to zoning codes that promote walkability at the street and neighborhood levels and more agencies that engage in cross-agency collaboration to provide transportation and recreation services.

##### Zoning Codes

Zoning codes, subdivision regulations, and building codes set the standards for the built environment. Our nationwide survey measured zoning codes that promote complete streets and mixed-use neighborhoods. Each code was measured as the percentage of community covered on a scale of 1 (0%), 2 (1–25%), 3 (26–50%), 4 (51–75%), and 5 (>75%). For example, “1” meant that the community did not have the zoning code, and 5 meant more than 75% of the community was covered by the zoning code.

Zoning codes at the street level promote complete streets and are the sum of four elements. Each community was rated on a scale of 4 to 20. A higher number meant that the community had more zoning codes that help promote complete streets. Each zoning code is shown in Table 3. More than half of respondents reported that 26–50% of their community was covered by zoning codes that mandate sidewalk systems, require street connections between adjacent developments, and contain pedestrian-friendly design guidelines, while less than 25% of their community was covered by codes that require complete streets (Table 3).

Zoning codes at the neighborhood level that promote mixed use are the sum of three elements (Table 3). More than half of the respondents indicated that more than 50% of their community had zoning to promote parks and recreation facilities in all neighborhoods, while zoning codes allowed mixed use in less than 25% of the community. Most communities did not provide a density bonus for affordable housing, open space, or transit. We expected that places with more zoning codes promoting complete streets and mixed use would have better built environment outcomes.

##### Cross-Agency Collaboration

Cross-agency collaboration helps link elements in the socioecological model to promote community health. Collaboration was measured by the number of agencies engaging in cross-agency partnerships. Among the four agencies measured, the public health department was the most common agency in the cross-agency partnership (44%), followed by the planning department (33%), parks and recreation department (25%), and transportation department (22%). Communities with more cross-agency collaboration were expected to have more transportation services and recreation and social activities.

### 3.3. Research Design

A generalized structural equation model (SEM) was run to test the theoretical framework in Figure 1. SEM was used to assess theoretical models by measuring the relationships in multivariate data and estimating models simultaneously [44,45]. Compared to multivariate regression, SEM provides more straightforward and integrative estimates of the coefficients [46]. Five regression models were run simultaneously. Linear regression and ordinal logit models were used based on the distribution of the dependent variables.

We used linear regression to measure the relations between individual, community, and physical activity (equations are given below).

Two linear regressions measured the relation between zoning codes and built environment:

(1) built environment (mixed-use neighborhood index) = f {zoning at neighborhood level, demographic factors, community controls, metro status},

(2) built environment (complete streets index) = f {zoning at street level, demographic factors, community controls, metro status}.

Two ordinal logit regressions measured the relation between cross-agency collaboration and services (transportation services, recreation and social activity):

(3) transportation services = f {cross-agency collaboration, community controls, metro status},

(4) recreation and social activity = f {cross-agency collaboration, community controls, metro status}.

Our final equation was our key dependent variable of interest and included all of the levels in our socio-ecological model.

(5) physical activity = f {built environment (street and neighborhood level), services (transportation services, recreation and social activity), perceptions of safety, demographic factors, metro status}.

Examining these factors as a structural system allowed us to model the theoretical framework by incorporating the physical environment, social and community context, the policy environment, and demographic factors into a comprehensive model of physical activity.

## 4. Results

We ran structural equation modeling in STATA 14.0 (StataCorp, College Station, U.S.) without latent factors [47]. Model results are shown in Table 4. The coefficients were standardized to compare the marginal effects between variables [47]. Results show that among all levels in the socio-ecological model, demographic factors and metro status had the greatest impact on physical activity (Equation (5) in Table 4). Compared to metro core places, suburban and rural communities had lower physical activity after controlling for other factors (rural: Std Coeff = −0.22, suburb: Std Coeff = −0.07). Poverty had the largest negative impact on physical activity (Std Coeff = −0.53). Other demographic factors also had a large negative impact, including percentage of minority population (Std Coeff = −0.18), percentage of population over age 65 (Std Coeff = −0.20), and average commuting time to work (Std Coeff = −0.10).

At the community level, the variables with the largest positive impact on physical activity were perceptions of safety (Std Coeff = 0.09), complete streets (Std Coeff = 0.08), and transportation services (Std Coeff = 0.09). Other community level factors also had a positive effect, but on a smaller scale, including mixed-use neighborhoods (Std Coeff = 0.06) and recreation programs and social activities (Std Coeff = 0.05). 

Our model results explored the direct effect of policy level (urban planning) on community development and the indirect effect of urban planning on physical activity. Local government efforts in planning, zoning, and cross-agency collaboration play an important role in building a better physical and social environment that contributes to a higher level of physical activity at the community level. Models of the built environment indices showed that zoning codes had the largest direct impact on built environments for both the mixed-use neighborhoods (Equation (1), Std Coeff = 0.36) and complete streets (Equation (2), Std Coeff = 0.56). Zoning codes also had an indirect impact on physical activity. Communities with more zoning codes promoting sidewalks, street connections and mixed use have a more active-friendly community environment that supports a higher level of physical activity. Cross-agency collaboration between departments of public health, planning, transportation, and parks and recreation had the largest impact on the number of transportation services (Equation (3), Std Coeff = 0.59) and recreation and social activities (Equation (4), Std Coeff = 0.54). Cross-agency collaboration also had an indirect effect on physical activity. Communities with more cross-agency collaboration had more transportation services and recreation and activities that facilitated a higher level of physical activity.

Community features also mattered for the physical and social environment. Denser and larger communities had more complete streets (Equation (2)) and transportation services (Equation (3)). Denser places had more mixed-use neighborhoods (Equation (1)) and social activities (Equation (4)), and smaller places had more social activities (Equation (4)). Older communities had more mixed-use neighborhoods (Equation (1)) and complete streets (Equation (2)). Suburban and rural communities had fewer transportation services (Equation (3)) compared to metro core areas, while the built environment indices were not differentiated by metro status, after controlling for other variables.

## 5. Discussion

**Figure 2:**
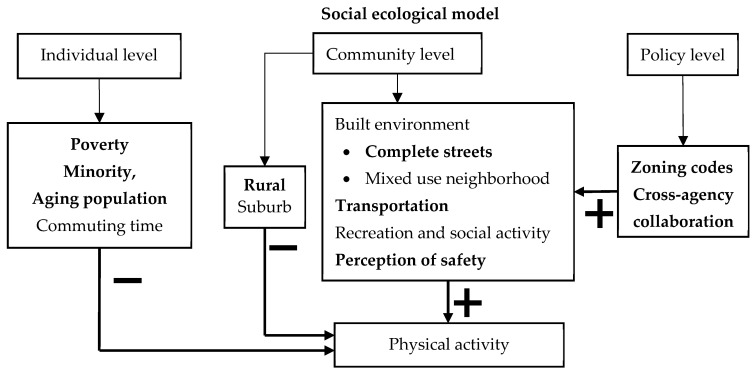
Model linking urban planning with physical activity to identify strategies for action. Source: Author analysis, adapted from Table 4. Note: “−“ denotes a negative relationship, “+” denotes a positive relationship.

Promoting physical activity is a growing concern among urban planners and public health advocates [10,13]. Figure 2 graphically shows our model results. While demographic factors are primary, the community and policy levels also play a role. Based on WHO’s (2019) action on physical activity [1], we unpacked the community level into built environment (mixed-use neighborhoods, complete streets), transportation services, recreation and social activity, and perceptions of safety. Urban planning was represented at the policy level, including zoning codes, and cross-agency collaboration. Our structural equation model linked the policy level (urban planning) with the community level in a socio-ecological model that helped us identify more strategies for building an active community. All factors listed in Figure 2 were significant. Those with a larger impact on physical activity are shown in bold.

Our models confirm that the strongest impacts on physical activity are from demographic factors: race, poverty, age, and commuting time (Figure 2). Although communities with a higher minority population had a lower level of physical activity, after controlling for other factors, our model results show that community features (built environment, transportation and recreation services) are not differentiated by race. Pairwise correlation analysis showed that a higher percentage of minority population is related to a higher poverty rate (r = 0.38, *p* < 0.05) and a longer average commuting time to work (r = 0.12, *p* < 0.05). This confirms other research that found the lower level of physical activity in minority communities could be related to the lack of access to transportation and recreation [16,48]. However, our models also suggest that lack of access could be related to lack of income and time. More planning attention should be given to relieving both income and time poverty. Strategies would include developing a broader array of transportation options that help reduce time lost to commuting, and promoting more recreation services to increase physical activity in these communities.

Both public health professionals and urban planners recognize the importance of the physical environment to health behaviors [1,3,32]. While mixed use and complete streets are built environments that support a higher level of physical activity, our models found that transportation services and perceptions of safety had a larger impact (Figure 2). While zoning supports better built environments, cross-agency collaboration helps communities offer more transportation and recreation services. Cross-agency collaboration has been recognized as critical to building a culture of health and affecting policy change [39,40]. Such collaboration helps communities provide more services and link the individual and community level in the ecological framework [9,38,49].

Recreation and social activities are also important for physical activity (Figure 2). Our models show cross-agency collaboration is a strategy communities can use to promote more transportation services and recreation services. While recreation programs were the most common social activity reported in our survey, other studies found that lower-income and minority communities often lack recreation services [50]. Thus, another strategy is to use cross-agency collaboration between public health, planning, transportation, parks and recreation departments to help increase recreation and social activities.

Safety is also a key factor. Our models show safety is as important as transportation and more important than the built environment. This effect holds for both urban and rural communities. Safety on streets, in neighborhoods, and in parks and playgrounds is important to encourage more physical activity. While traffic safety is a primary concern in urban communities, fear of crime has been shown to be a barrier to physical activity in rural communities [27,28]. In fact, rural communities have higher crime and are less likely to have community policing programs [51]. To encourage physical activity, another strategy is for planners to give more attention to safety concerns.

Rural communities have a lower level of physical activity, due in part to higher poverty and a higher percentage of older adults. Transportation services can improve residents’ access to health care, fresh food, and opportunities for physical activity [32,33,39,52]. Flexible transportation services are especially important in building an active rural community [53]. While public transit is often lacking in rural communities, our survey found that school buses and volunteer driver programs are equally common in rural and urban communities and demand–response transit is more common than public transit in rural communities. Thus, a range of transportation services are an important strategy for promoting physical activity.

The CDC recommends mixed-use neighborhoods and walkable communities to promote more physical activity [3]. Although our results show these features are related to a higher level of physical activity, many rural communities lack these physical built environments. Mixed-use neighborhoods and complete streets are difficult to create in the short term, but recreation and social activities and transportation services can be addressed in the short term. Despite the CDC’s focus on active transportation as a strategy to promote more physical activity [3], this may not be the best way to encourage more physical activity in rural communities. Providing more recreation programs and a safe environment may be more effective strategies to encourage people living in rural communities to become more active.

This study has limitations. First, the measure of physical activity was constructed from self-reported data, and there could be response bias [54]. Second, although multiple years of data were used in analysis, this study only showed the relations between urban planning, community environment, demographic factors and leisure time physical activity. Causal relation should not be interpreted from this study. Future study could consider the time-lag effect of community environment on physical activity. Third, this study was within the context of the US. Future study could utilize the research framework in other contexts and test factors at the individual, community, and policy levels that are related to physical activity.

## 6. Conclusions

This study linked the individual, community, and policy layers in the socio-ecological model to explore factors related to physical activity. It contributes to the literature by bringing urban planning into the socio-ecological model and building a structural equation model to explicitly measure the impact of the policy level (zoning, cross-agency collaboration) on community-level physical and social environments. This provides new insights on community strategies to promote physical activity.

Demographic factors create challenges to improving physical activity in communities with a higher poverty rate, larger minority population, aging population, and a longer commuting time to work. While most demographic factors cannot be changed, planners can help address time constraints and promote transportation and recreation services that enhance access to physical activity.

Both public health and urban planning focus on interventions in the physical environment (zoning, mixed use) [3,32], but we found that the social environment, including recreation and social activities and perceptions of safety, are also important. For many rural or minority communities that lack physical environments supportive of active transportation, planners can focus on promoting more transportation options and recreation and safety programs.

Policy is recognized as an important level to promote public health and address health disparities [30], but it is difficult to measure, especially at the community level due to lack of data. We conducted the first nationwide survey of community policy factors to measure local government efforts in planning and zoning and cross-agency collaboration. We found that addressing the built environment is not enough; attention also must be given to transportation services, recreation programs and safety. While planning and zoning codes help create more active-friendly built environments, cross-agency collaboration helps promote complete streets, transportation services, and recreation and safety. By linking the individual, community and policy layers, our comprehensive model helps differentiate physical activity across places. Our analysis provides insights on the role both planners and public health leaders can play in promoting physical activity, especially in rural and minority communities where residents face greater income, time and age constraints. A comprehensive approach is needed; complete streets, transportation options, recreation and safety all play a role.

## Figures and Tables

**Table 1:** Descriptive statistics, US municipalities.

	Obs	Mean	Std. Dev.	Min	Max
Physical activity (%) ^1^	1312	75.52	5.87	55.5	89.8
**Individual level: demographic factors**					
Poverty rate (%) ^3^	1312	12.62	7.55	0.37	48.35
Percentage of minority population ^3^	1312	26.68	22.57	0.12	99.62
Average commute time to work (minutes) ^3^	1312	24.67	6.14	8	46
Percentage of population over age 65 ^3^	1312	17.40	5.78	3.20	57.65
**Community level**					
***Physical and social environment***					
Built environment—complete streets index (3 elements, scale 1–5) ^2^	1207	7.18	2.67	3	15
Built environment—mixed use neighborhood index (4 elements, scale 1–5) ^2^	1217	11.37	4.15	4	20
Transportation services (4 elements) ^2^	1312	1.45	1.03	0	4
Recreation and social activity (4 elements) ^2^	1312	1.64	1.31	0	4
Perceptions of safety (2 elements, scale 1–5) ^2^	1215	7.94	1.66	2	10
***Metro status***					
Metro core ^4^	1312	0.18	0.38	0	1
Suburb ^4^	1312	0.52	0.50	0	1
Rural ^4^	1312	0.31	0.46	0	1
***Community controls***					
Population (ln) ^3^	1312	9.94	1.46	6.05	15.19
Population density (ln) ^3^	1312	6.17	2.06	−0.81	10.88
Median age of housing ^3^	1301	42.44	13.26	8	79
**Policy level: urban planning**					
Zoning—street level index (4 elements, scale 1–5) ^2^	1131	11.96	5.41	4	20
Zoning—neighborhood level index (3 elements, scale 1–5) ^2^	1149	8.23	2.98	3	15
Cross-agency collaboration (4 elements) ^2^	1312	1.55	1.29	0	4

Data sources: ^1^ CDC 500 Cities & PLACES Data Portal 2021 [14] ^2^ Planning for All Ages Survey 2019 ^3^ American Community Survey 2015–2019 [15] ^4^ US Census Delineation files 2018 [43].

**Table 2:** Built environment measures.

Built Environment: What Percentage of Your Community Contains the Following? (% of Respondents Reporting)	0% (1)	1–25% (2)	26–50% (3)	51–75% (4)	>75% (5)
**Complete streets index**					
Sidewalk system connecting residences and services	9	33	19	17	22
Bike lanes	34	49	9	5	3
Streets designed for all modes of transit—walking, biking, etc.-not only cars	31	44	10	6	8
**Mixed-use neighborhood index**					
Park or playground within half-mile of every resident	8	33	20	17	21
Public gathering spaces	7	39	22	15	17
A mix of retail, services, and housing	9	40	22	12	17
Fresh food markets	19	47	14	8	13

Data source: US municipalities, N = 1312. Planning for All Ages survey, 2019.

**Table 3:** Zoning code measures.

Zoning Codes: In What Percentage of Your Community Do Zoning Codes Do the Following? (% of Respondents Reporting)	0% (1)	1–25% (2)	26–50% (3)	51–75% (4)	>75% (5)
**Street-level index**					
Mandate sidewalk system	24	18	10	8	40
Require street connections between adjacent developments	22	21	9	11	37
Contain pedestrian-friendly design guidelines	23	26	12	10	29
Require “complete streets”	39	22	9	9	21
**Neighborhood-level index**					
Promote parks or recreation facilities in all neighborhoods	14	18	14	16	38
Allow mixed use	11	39	25	12	13
Provide density bonuses	52	23	9	5	11

Data source: US. municipalities, N = 1312. Planning for All Ages survey, 2019.

**Table 4:** Model results—generalized SEM (standardized coefficients).

	Built Environment			Services		Physical Activity (Equation (5)) ^1^
	Mixed-Use Neighborhood (Equation (1)) ^2^	Compete Streets (Equation (2)) ^2^		Transportation Services (Equation (3)) ^2^	Recreation and Social Activity (Equation (4)) ^2^	
**Policy Level**						
Zoning—neighborhood level Index ^2^	0.36 **					
Zoning—street level index ^2^		0.56 **				
Cross-agency collaboration ^2^				0.59 **	0.54 **	
**Community Level**						
***Community controls***						
Population (ln) ^3^	0.06	0.13 **		0.26 **	−0.13 **	
Population density (ln) ^3^	0.37 **	0.12 **		0.37 **	0.47 **	
Age of housing ^3^	0.11 **	0.12 **				
***Metro status***						
Suburban ^4^	−0.07	−0.02		−0.23 **	−0.17 **	−0.07 *
Rural ^4^	0.05	0.06		−0.24 **	−0.16 *	−0.22 **
***Physical and social environment***						
Built environment—mixed-use neighborhood index ^2^						0.06 *
Built environment—complete streets index ^2^						0.08 **
Transportation services ^2^						0.09 **
Recreation and social activity ^2^						0.05 *
Perceptions of safety ^2^						0.09 **
**Individual Level: Demographic factors**						
Poverty rate ^3^	−0.07 *	−0.02		−0.01	0.04	−0.53 **
Percentage of minority population ^3^	0.02	0.04		−0.05	0.06	−0.18 **
Percentage of population over 65 ^3^	−0.01	−0.02		0.17 *	−0.01	−0.20 **
Average commuting time to work ^3^						−0.10 **
Log likelihood	−11,416.307					

N = 1312 US municipalities. Data sources: ^1^ CDC 500 Cities & PLACES Data Portal 2021 [14] ^2^ Planning for All Ages Survey 2019 ^3^ American Community Survey 2015–2019 [15] ^4^ US Census Delineation files 2018 [43]. Note: * *p* < 0.05, ** *p* < 0.01.

## Data Availability

Survey data are unavailable due to privacy restrictions. Data on physical activity, community demographic factors, and community controls are publicly available. See data sources for detailed information.
